# Prevalence of Metabolic Syndrome and Association with Physical Activity and Frailty Status in Spanish Older Adults with Decreased Functional Capacity: A Cross-Sectional Study

**DOI:** 10.3390/nu14112302

**Published:** 2022-05-30

**Authors:** Jorge Subías-Perié, David Navarrete-Villanueva, Ángel Iván Fernández-García, Ana Moradell, Eva Gesteiro, Jorge Pérez-Gómez, Ignacio Ara, Germán Vicente-Rodríguez, José Antonio Casajús, Alba Gómez-Cabello

**Affiliations:** 1 GENUD (Growth, Exercise, Nutrition and Development) Research Group, University of Zaragoza, 50009 Zaragoza, Spain; jsubias@unizar.es (J.S.-P.); dnavarrete@unizar.es (D.N.-V.); angelivanfg@unizar.es (Á.I.F.-G.); amoradell@unizar.es (A.M.); gervicen@unizar.es (G.V.-R.); joseant@unizar.es (J.A.C.); 2 Instituto Agroalimentario de Aragón (IA2), University of Zaragoza, 50013 Zaragoza, Spain; 3 Instituto de Investigación Sanitaria Aragón (IIS Aragón), 50009 Zaragoza, Spain; 4 Department of Physiatry and Nursery, Faculty of Health Sciences, University of Zaragoza, 50009 Zaragoza, Spain; 5 Red Española de Investigación en Ejercicio Físico y Salud, EXERNET, University of Zaragoza, 50009 Zaragoza, Spain; eva.gesteiro@upm.es (E.G.); jorgepg100@gmail.com (J.P.-G.); ignacio.ara@uclm.es (I.A.); 6 Department of Physiatry and Nursery, Faculty of Health and Sport Sciences, University of Zaragoza, 22002 Huesca, Spain; 7 ImFine Research Group, Departamento de Salud y Rendimiento Humano, Facultad de Ciencias de la Actividad Física y del Deporte-INEF, Universidad Politécnica de Madrid, 28040 Madrid, Spain; 8 HEME (Health, Economy, Motricity and Education) Research Group, Faculty of Sport Sciences, University of Extremadura, 10003 Caceres, Spain; 9 GENUD Toledo Research Group, Universidad de Castilla-La Mancha, 45071 Toledo, Spain; 10 Centro de Investigación Biomédica en Red Fragilidad y Envejecimiento Saludables(CIBERFES), Instituto de Salud Carlos III, 28029 Madrid, Spain; 11 Centro de Investigación Biomédica en Red Fisiopatología de la Obesidad y Nutrición (CIBERObn), Instituto de Salud Carlos III, 28029 Madrid, Spain; 12 Centro Universitario de la Defensa, University of Zaragoza, 50090 Zaragoza, Spain

**Keywords:** health, metabolic syndrome, cardiovascular diseases, older populations, exercise

## Abstract

Metabolic syndrome (MetS) is a cluster of medical conditions associated with several health disorders. MetS and frailty can be related to prolonged physical deconditioning. There is a need to know whether there is concordance between the different ways of diagnosing it and to know their prevalence in Spanish older adults. Thus, the aims of this study were to describe the prevalence of MetS; to analyse the concordance between different definitions to diagnose MetS; and to study the associations between MetS, frailty status, and physical activity (PA) in older adults with decreased functional capacity. This report is a cross-sectional study involving 110 Spanish older adults of ages ≥65 years with decreased functional capacity. Clinical criteria to diagnose MetS was defined by different expert groups. Anthropometric measurements, blood biochemical analysis, frailty status, functional capacity, and PA were assessed. The Kappa statistic was used to determine the agreement between the five MetS definitions used. Student’s *t*-test and the Pearson chi-square test were used to examine differences between sex, frailty, and PA groups. The sex-adjusted prevalence of MetS assessed by the National Cholesterol Education Program—Third Adult Treatment Panel was 39.4% in men and 32.5% in women. The International Diabetes Federation and the Harmonized definitions had the best agreement (k = 1.000). The highest odds ratios (ORs) of cardiometabolic risk factors to develop MetS were elevated triglycerides (37.5) and reduced high-density lipoprotein cholesterol (27.3). Central obesity and hypertension prevalence were significantly higher in the non-active group (70.7% and 26.8%, respectively), compared to the active group (50.0% and 7.7%, respectively). Moreover, the active group (OR = 0.85, 95% CI = 0.35, 2.04) and active women group (OR = 0.77, 95% CI = 0.27, 2.20) appeared to show a lower risk of developing this syndrome. MetS is highly prevalent in this sample and changes according to the definition used. It seems that sex and frailty do not influence the development of MetS. However, PA appears to decrease central obesity, hypertension, and the risk of developing MetS.

## 1. Introduction

Populations worldwide are living longer. Between 2015 and 2050, the percentage of the world’s population aged over 60 years will nearly double from 12% to 22% [1]. The ageing process of the human population is known as a challenge but also as a risk factor for chronic illness. Increasing life expectancy associated to chronic diseases will increase vulnerability to frailty, senile dementia, functional decline, and incapacity [2]. One of the most prevalent chronic diseases associated to ageing is metabolic syndrome (MetS). Therefore, the prevalence of this syndrome will increase in the global population enlarging its relevance in the health system [1,2].

MetS is a cluster of medical conditions that includes central obesity, hypertension dyslipidemia, and insulin resistance [3]. It is known to be associated with several disorders such as type 2 diabetes mellitus (T2DM), cardiovascular disease (CVD), cancer, gout and asthma [4,5,6]. People with MetS have a 2-fold increased risk of developing CVD and a 5-fold increased risk of developing T2DM than those without MetS [7,8]. The underlying causes of the MetS remain a challenge for the experts, but both elevated glucose and central obesity are considered primary factors [9]. Genetics, physical inactivity, ageing, oxidative stress, a proinflammatory state, and hormonal changes may also have a causal effect, but their role may vary depending on different factors such as sex, age, and ethnic group, among others [10,11].

Clinical criteria for diagnosing MetS were developed by a number of expert groups. The most widely accepted were designed by the World Health Organization (WHO) [12]; the National Cholesterol Education Program—Third Adult Treatment Panel (NCEP-ATP III) [13]; the American Heart Association (AHA)/National Heart, Lung and Blood Institute (NHLBI) [14]; the International Diabetes Federation (IDF) [15]; and Harmonized Definition [16]. Specifically, NCEP-ATP III is one of the most widely used in MetS studies [16]. All groups agreed with the five components of the MetS, however, the existing guidelines are either difficult to use or give conflicting results when attempting to identify individuals with the MetS in clinical practice [10,17]. It seems that the prevalence of MetS changes according to the diagnostic criteria used [17], and the sex [17,18], age [19,20], and ethnicity of the population analysed [21,22]. Despite the different definitions, MetS shows a rising prevalence in the population, which is ascribed mainly to increasing obesity and sedentary lifestyles. As a result, this syndrome is now both a public health and a clinic problem. A deep knowledge of the agreement between different diagnostic criteria or even which one best describes a riskier environment in older people can help us to accurately describe the problem and to accurately analyse the effects of treatment or prevention measures in a large and special population group.

It is now well established that MetS and frailty are associated with negative outcomes including decline of functional capacity, falls, clinical depression, senile dementia, and increased rate of mortality [2]. However, the association of frailty status with MetS remains unclear. The WHO defines frailty as “a progressive age-related decline in physiological systems that results in decreased reserves of intrinsic capacity, which confers extreme vulnerability to stressors and increases the risk of a range of adverse health outcomes” [18]. In the same way, it is known that biological factors are one of the major risk factors for age-related diseases, being chronological-age moldable. The relationship between frailty and MetS is bidirectional, and they are intimately associated, even in younger subjects. There are several factors that can promote anabolic resistance and muscle degradation, such us inflammatory processes, oxidative stress, insulin resistance, cytokine changes, and ageing. Furthermore, insulin resistance is further altered by decreased muscle mass. This cycle continues until pancreatic beta-cells do not cover the demands of the organism. Even when muscle mass may not be decreased in obese older adults, the quality of muscle could be affected due to fat infiltration resulting in reduced muscle strength and function [2,11].

Likewise, according to WHO 2020 guidelines, the benefits of physical activity (PA) in the adult population are observed within average weekly volumes of 150–300 min of moderate intensity or 75–150 min vigorous intensity, or an equivalent combination [19]. Both observational and interventional studies propose an important role for PA in mitigating MetS in adults [20]. It seems that raising the total amount of PA in the sedentary and overweight population has beneficial effects on metabolic risk [21]. Another study suggests that those men who meet the PA recommendations have less risk to develop MetS than their sedentary equivalents [22]. The role of PA in fitness is well known, but to the best of our experience and information, the influence of PA on cardiovascular, lipid, and glucose disorders in older adults with decreased functional capacity has not been studied. As decreased functional capacity is a risk factor for frailty, assessing the association between decreased functional capacity and MetS risk will be a useful tool in designing more precise and successful frailty prevention strategies.

Some studies have described the prevalence of MetS in adults and older adults [3,23,24,25,26,27,28]. However, most of them have focused on the Chinese population [26,27,28] or people with a specific pathology such as T2DM [3]. To the best of our knowledge, no other studies have examined MetS in older adults with decreased functional capacity from the Spanish population. Moreover, the main aims of this study were: (1) to describe the prevalence of the individual components and general diagnosis of MetS; (2) to analyse the concordance between the different criteria for MetS; (3) to know the association between MetS and frailty; and (4) to know the association between MetS and PA level, in a sample of Spanish older adults with decreased functional capacity.

## 2. Materials and Methods

### 2.1. Study Design and Participants

This cross-sectional study was carried out with data from the participants of the EXERNET-Elder 3.0 project (2018–2020) [29]. One of the aims of this project was to improve the physical functioning of older adults with a decreased functional capacity through a multicomponent exercise program. The researchers initially recruited169 older adults, but in some participants not all measures were recorded to assess MetS, or they dropped out of the research due to worsening health status. Finally, 110 participants were included in the study with all measures recorded to assess the metabolic risk. Participants were recruited from 3 neighbourhood’s from Zaragoza, Spain, with different economic and social characteristics to have a representative sample. Specifically, 4 primary healthcare centers and 3 nursing homes were involved in this project. Finally, in order to be included, participants had to fulfil the following criteria: (a) be older than 65 years; (b) have a decreased functional capacity (≤9 points) in relation to the cut-off points of the short physical performance battery [30,31]; (c) not suffer dementia and/or cancer. The complete methodology of the physical intervention is detailed in full by Fernandez–Garcia, et al. [29].

The health outcomes were compiled with personal interviews using a structured questionnaire, followed by physical analysis to evaluate anthropometrics, body composition, functional capacity [30], frailty status [32], and PA data according to objective and standardized protocols. Moreover, a biochemical analysis was performed to analyse the level of high-density lipoprotein cholesterol (HDL-C), triglycerides (TGs), and glucose. Also, blood pressure (BP) was measured to learn the systolic blood pressure (SBP) and diastolic blood pressure (DBP). The complete set of project variables is available elsewhere [29]. This research was enrolled in the electronic repository clinicaltrials.gov (accessed on 12 February 2018, reference number: NCT03831841).

### 2.2. Ethics Statement

The entire sample of this study received oral and written information about the aims, possible benefits, and risks derived from participation in this physical exercise programme. Afterwards, all the included participants obtained and signed the written informed consent. The research was performed in accordance with the Ethical Guidelines of the Declaration of Helsinki of 1961, revised in Fortaleza (2013) [33], and the current legislation of human clinical research of Spain (Law 14/2007). The study protocol was certified by the Hospital Universitario Fundación Alcorcón Ethics Committee (16/50).

### 2.3. Diagnosis of Metabolic Syndrome

A number of expert groups have described clinical criteria to diagnose MetS. In this study, we have used the 5 main methods to diagnose MetS, summarized in Table 1. It seems that Harmonized and NCEP-ATP III criteria were primarily used, followed by the AHA/NHLBI and IDF definition [17]. Specifically, the agreement in diagnosis of MetS, the odds ratios (ORs) of individual components of MetS, and the association between MetS, PA, and frailty status were calculated using NCEP-ATP III, as it is the most commonly used in the literature consulted.

### 2.4. Biochemical Parameters

All participants were fasting up to the blood test, and it was carried out through venous puncture on the cephalic or median antecubital vein using a 21-gauge butterfly needle (BD Vacutainer Safety-Lok., BD Biosciences, North Ryde, NSW, Australia) with four different 4 mL Vacutainer^®^ tubes (BD Biosciences, North Ryde, NSW, Australia), one containing citrate and three containing ethylenediaminetetraacetic acid. Blood samples for biochemical analysis were allowed to clot for at least 60 min. Then, the blood samples were centrifuged for 10 min and 3500 rpm, and serum was collected. The biochemical analysis of this serum was analysed within 24 h after collection, and the main parameters analysed in this study were glucose, TGs, and HDL-C.

### 2.5. Blood Pressure

The measurement protocols of the European Society of Cardiology and the European Society of Hypertension have been followed to measure the BP [34]. BP was taken without having taken antihypertensive drugs on the measurement day. It was measured at the sitting position using a calibrated digital Omron Automatic BP Monitor (OMRON M6 COMFORT IT [HEM-7322U-E] Kyoto, Japan), with an appropriately sized cuff on the left or right arm after taking rest for at least 3–5 min. All BP measurements were taken twice by two nurses, with 1–2 min intervals. The mean values of both measurements were calculated for SBP and DBP.

### 2.6. Body Composition and Anthropometric Measurements

Body weight (kg) and body fat (%) were the main variables of body composition included in this study. To measure these outcomes, a Bio-Electrical Impedance Analysis machine with a 200 kg maximum capacity and 50 g error margin (TANITA BC-418MA, Tanita Corp., Tokyo, Japan) was used. Participants removed their shoes and heavy clothes before weighing. All measurements were performed at the same hour, with an empty bladder and the same conditions for all participants and evaluations. Body mass index (BMI) was estimated by dividing weight (kg) by squared height (m^2^).

Height (cm) and waist circumference (WC; cm) were registered following the International Society for the Advancement of Kinanthropometry (ISAK) protocol [35]. Two researchers authorized as ISAK level 1 anthropometrists compiled the anthropometric measurements. A portable stadiometer with 2.10 m maximum capacity and 1 mm error margin (Seca, Hamburgo, Germany) was used to measure height. Subjects stood barefoot with their scapula, buttocks and heels resting against a wall; the neck was held in a natural non-stretched position; and the head was in Frankfort’s plane.

WC was taken at the level of the narrowest point between the lower costal border and the iliac crest. In case it was not appreciated, measure was taken between these points. Hip circumference was measured at the level of the greatest posterior protuberance of the buttocks. A flexible non-elastic measuring tape Rosscraft Anthrotape (Rosscraft Innovations Inc., Vancouver, BC, Canada) was used to take WC and hip circumference. Measures were performed twice, and the mean was calculated. If there were incongruences between the first two evaluations, a third measure was done, and the median was calculated. The waist-to-hip ratio is indicative of regional fat distribution, and it was determined using the formula as WC divided by hip circumference.

### 2.7. Frailty Status

The Frailty Phenotype of Fried was used to assess the frailty status. This test is made of 5 items: unintentional weight loss (>4.5 kg in the year before or at least 5% of body weight), self-reported exhaustion (felt especially tired during the last week), weakness (low grip strength), slow usual gait speed (4.5 m), and low PA (less than 2 h walking per week). When 3 or more of these items were met, the degree of frailty was reached (frail group [FRA]), while only 1 or 2 items denoted pre-frailty (pre-frail group [PREF]), and people with 0 items made up the robust group (ROB) [32].

### 2.8. Physical Activity

Wrist-worn triaxial accelerometers (GENEActiv Active insights Ltd., Cambridges, UK) were used to register PA. The device was worn on the nondominant wrist. These accelerometers obtained data for 1 week at a frequency of 10 Hz, which is enough to categorize regular activities [36]. To include participants in the statistical analysis, they must have worn the accelerometer for at least 4 valid days including at least 480 min (8 h per day). Triaxial data was condensed in 1 vector, calculating the Euclidean norm minus 1 to isolate human movement from gravitational acceleration [37], and aggregated into 60 s epochs. Each epoch was classified as either light PA, or moderate to vigorous PA (MVPA) time, according to previously defined cut-off points [38] which are specific for this population and accelerometer location and that have been designed to optimize both sensitivity and specificity of classification [39]. To make a distinction between inactivity periods during the day and sleep time, the sleep period time window was detected [40]. Through accelerometer data we divided the total sample into 2 groups: the active and non-active group. Following the WHO 2020 guidelines on PA [19], the Active group was the one that accumulated at least 150 min of MVPA per week.

### 2.9. Statistical Analysis

All analyses were performed using the Statistical Package for the Social Sciences v. 25.0 for Windows (IBM SPSS, Inc., Chicago, IL, USA) with statistical significance set at level *p* < 0.05 in all tests. Shapiro–Wilk test was used to prove the normality of the sampling distribution, all variables had a normal distribution, and therefore parametric test were used.

Sample sizes were calculated for estimating proportion with a confidence level of 95%, a sampling error (α) of 0.05, and a Z value (Z α) of 1.96. Therefore, it was estimated that a minimum of 189 participants would be needed for final sample.

Continuous variables were presented as mean values ± standard deviation. Categorical variables were presented as frequencies (%). Sex-specific prevalence of MetS was calculated using the WHO, NCEP-ATP III, AHA/NHLBI, IDF, and Harmonized definitions. The agreement and disparity in diagnosis of MetS, the ORs of individual components of MetS, and the association between MetS, PA, and frailty status were calculated using NCEP-ATP III criteria. Student’s *t*-test and the Pearson chi-square test were used to examine differences in continuous and categorical variables, respectively, between frailty groups. Crosstabs were used to calculate the ORs of individual components of MetS with a 95% confidence interval. Chi-square tests were used to analyse independent associations between MetS, habitual PA, and frailty. Data are presented as prevalence, degrees of freedom (χ^2^), and p value.

The concordance between the five definitions of MetS was analysed by the Kappa (*k*) statistic. The Kappa value is between 0 and 1, where the value of 1 indicates perfect agreement and values less than 1 involve poorer agreement. In particular, a *k* index less than 0.20 implies a weak agreement, 0.21 to 0.40 represents as fair agreement, 0.41 to 0.60 is interpreted as moderate agreement, 0.61 to 0.80 means good agreement, and 0.81 to 1 suggests a very good agreement.

## 3. Results

The details regarding socio-demographic, frailty status, body composition and anthropometric measurements, and serum biochemical and BP characteristics of participants are presented in Table 2.

### 3.1. Prevalence of Metabolic Syndrome

The prevalence of single components of MetS and MetS defined by the different groups of experts in Spanish older adults with decreased functional capacity is presented in Table 3. Total prevalence of MetS ranged from 22.2% to 39.4% in men and from 17.5% to 39% in women, depending on the definition. In this sample, statistically insignificant differences were found between women and men in the total prevalence of MetS, elevated glucose, hypertension, elevated TGs, and reduced HDL-C. Regarding individual components of MetS, central obesity was the most prevalent while hypertension was the least prevalent, but 60% of the sample was being treated with an antihypertensive drug. Prevalence of central obesity by sex was different depending on the diagnostic criteria: it was more prevalent (*p* < 0.001) in men than in women according to the WHO definition, while it showed a higher prevalence in women than in men (*p* < 0.001) based on the IDF and Harmonized definitions.

### 3.2. Agreement and Disparity in Diagnosis of Metabolic Syndrome

The concordance as well as sensitivity and specificity of the MetS for the referenced definitions are shown in Table 4. The *k* index statistics confirmed a significant agreement between the definitions. The best agreement observed was between the IDF and Harmonized definitions (*k* index = 1.000). The NCEP-ATP III and AHA/NHLBI criteria had the second-best agreement with the highest *k* index. The IDF and Harmonized criteria had the third-best agreement. The WHO and NCEP-ATP III criteria had the worst agreement with the lowest *k* index. All definitions showed good specificity. The NCEP-ATP III, AHA/NHLBI, IDF, and Harmonized criteria presented 100% of sensitivity, while WHO criteria showed a poor sensitivity.

### 3.3. Odds Ratios of Individual Components of Metabolic Syndrome

Table 5 shows the ORs of individual components of MetS to develop MetS based on NCEP-ATP III criteria. Of the five MetS components, elevated TGs and reduced HDL-C showed the highest ORs, while hypertension showed the lowest.

### 3.4. Association between Frailty Status and Metabolic Syndrome

**Figure 1:**
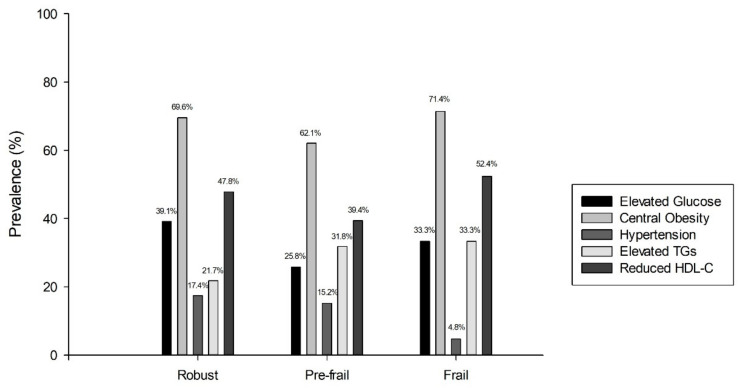
Prevalence of MetS cardiometabolic risk factors by frailty group. HDL-C, high-density lipoprotein cholesterol; TGs, triglycerides.

Although no statistically significant differences were found in the prevalence of MetS between frailty groups, the ROB group showed a higher prevalence at 39.1% (*n* = 23, 11 women; 78.4 ± 6.5 y), followed by 33.3% (*n* = 21, 17 women; 81.1 ± 6.5 y) in the FRA group and 33.3% (*n* = 66, 49 women; 81.3 ± 5.3 y;) in the PREF group. Moreover, Figure 1 shows the prevalence of individual MetS components across the frailty status. There were no statistically significant differences in cardiometabolic risk factors between frailty groups.

### 3.5. Association between Physical Activity Behaviour and Metabolic Syndrome

**Figure 2:**
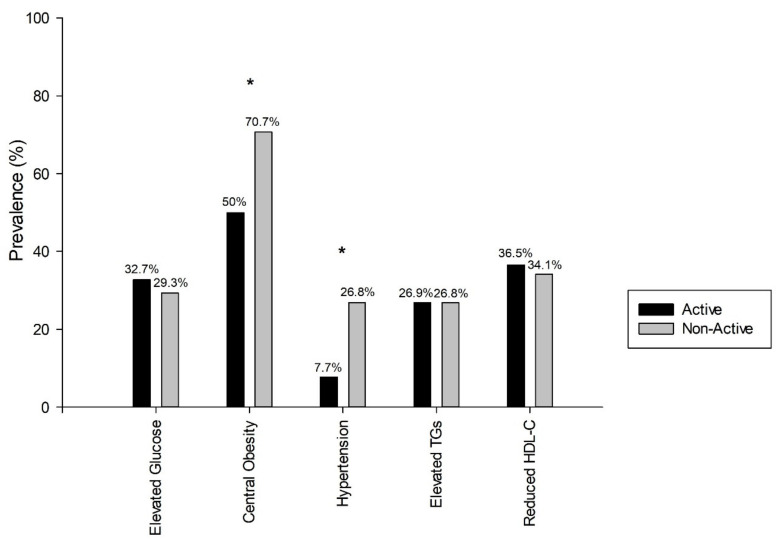
Prevalence of MetS cardiometabolic risk factors by physical activity groups. HDL-C, high-density lipoprotein cholesterol; TGs, triglycerides. * Statistically significant differences (*p* < 0.05 *). Pearson chi-square test.

The PA data from 93 participants were considered in this section. Through accelerometer data, we divided the total sample into 2 groups, the active group (*n* = 52, 37 women; 78.3 ± 4.8 y) and non-active group (*n* = 41, 30 women; 83.1 ± 6.1 y). There were no significant differences in the prevalence of MetS between PA groups. Figure 2 shows the prevalence of MetS components across the PA groups. Central obesity was significantly higher in non-active group at 70.7% (*n* = 29) compared to the active group at 50.0% (*n* = 26, χ^2^ = 4078, *p* = 0.043). Hypertension prevalence was significantly higher in the non-active group at 26.8% (*n* = 11) compared to the active group at 7.7% (*n* = 4, χ^2^ = 6,206, *p* = 0.013). However, there were no significant differences in the presence of reduced HDL-C, elevated glucose, and TGs between groups.

Moreover, the ORs of having MetS were 0.85 (95% CI = 0.35, 2.04) in the whole active group. Specifically, the active women group had ORs of 0.77 (95% CI = 0.27, 2.20) and the active men group showed ORs of 3.04 (95% CI = 0.57, 16.19) to development of this syndrome.

## 4. Discussion

In this cross-sectional study of Spanish older adults with decreased functional capacity, the prevalence of MetS and the prevalence of individual components of MetS has been described according to five different definitions for MetS. Moreover, the study has analysed the concordance between the different definitions to diagnose MetS and the ORs of individual components of MetS to develop this syndrome in this sample. Furthermore, the association between MetS, frailty, and PA has been evaluated in this research.

### 4.1. Prevalence of MetS

As was pointed out in the results section, the prevalence of MetS in this sample depended on the definition. The WHO definition showed 15.7% of people with MetS, followed by NCEP-ATP III and AHA/NHLBI with 34.5%, and the IDF and Harmonized definitions with 37.3%. The diagnostic criteria used may explain the dissimilar magnitudes of MetS prevalence. Firstly, a higher cut-off value for glucose in the WHO and NCEP-ATP III definitions compared to the AHA/NHLBI, IDF, and Harmonized definitions leads to the inclusion of participants with a relatively lower level of this risk factor, giving a higher prevalence of MetS with the last definitions. Secondly, the WC criterion in the IDF and Harmonized definitions used a lower cut-off value compared to NCEP-ATP III and AHA/NHLBI, giving a higher prevalence of MetS with the IDF and Harmonized definitions. Thirdly, the WHO criteria have a higher cut-off value to diagnose hypertension and a lower cut-off value for reduced HDL-C compared with rest of the definitions used, giving a lower prevalence of MetS than the others when considering this definition. These differences could explain why the WHO criteria obtained the lowest prevalence of MetS.

Several reports have shown prevalence of MetS in different age groups and sexes. Moreira et al. [23] estimated that around 20–25% of the world’s adult population have MetS. Higuita–Gutierrez et al. [24] observed that the prevalence of MetS in adults under 65 years of age was 35.4%. Meanwhile, Barranco–Ruiz et al. [41] noted a prevalence of 54.9% in people over 60 years, with a higher prevalence among females than males (59.8% vs. 47.3%, respectively). Ervin et al. [25] observed that people aged between 40–59 years were about 3 times as likely as those aged 20–39 years to meet the criteria for MetS. Furthermore, males 60 years and over were more than 4 times as likely and females 60 years and over were more than 6 times as likely as the youngest age group to meet the criteria. Moreover, one study reported that ethnicity could modify the prevalence of MetS, but this variable was not analysed in this study [25].

In our study, it seems that the prevalence of MetS was higher in women than in men when the prevalence was analysed with the AHA/NHLBI, IDF, and Harmonized definitions. This may be explained by the higher prevalence of abdominal obesity, elevated TGs and low HDL-C in women than in men. However, it appears that a higher prevalence of glucose and hypertension in men does not report a higher prevalence of MetS. Sex differences are consistent with other Chinese population studies [26,27,28]. As is commonly known, MetS is more prevalent as age increases. In those studies in which the sample is older than 65 years, there is a higher prevalence of MetS [26] than in those with younger participants [27,28].

### 4.2. Prevalence of Individual Components of Metabolic Syndrome

There are important differences in the results of the prevalence within individual components of MetS depending on each study. Central obesity (59.1%) and reduced HDL-C (34.5%) were the most common MetS component in our study. Similar results were obtained in a cross-sectional study that analysed the prevalence of Mets in a sample of 15,540 Chinese adults in which reduced HDL-C (33.9%) was the second most common MetS component, but hypertension (41.2%) was the most common [28]. Another cross-sectional study of 8,814 US population obtained the same results as in the present study, with central obesity (38.6%) being the most common and reduced HDL-C (37.1%) the second most common MetS component [42]. Moreover, an observational study with 64,902 Chinese adults observed that central obesity (50.1%), followed by increased TGs (49.5%) and hypertension (46.8%), were the most prevalent individual components of MetS [43]. However, other studies performed with Chinese adults, observed that hypertension was the most common [26,27,44], while in the present study it was the least prevalent. It is important to remember that although the participants did not take the drug on the day of the BP measurement, 60% of the participants were on treatment with an antihypertensive drug. Therefore, it seems that the prevalence of hypertension would be higher if pharmacological treatment were deleted, but the most prevalent component would still be central obesity. Consequently, it seems that the age, ethnicity, and cultural customs of each country can modify the prevalence of individual components of MetS, but there is no single factor that explains these differences.

### 4.3. Agreement and Disparity in Diagnosis of Metabolic Syndrome

In our study, an excellent agreement was observed between the IDF and Harmonized definitions. This is because the five components agree in both definitions, but there is a change in the general definitions to diagnose MetS. The other difference is that the Harmonized definition includes a specific ethnic cut-off point for WC. Our findings were similar from an observational study with Chinese adults participants that obtained the best agreement (*k* = 0.708) between these definitions [43], and a study with Ethiopian T2DM adults participants (*k* = 0.650) [45]. Moreover, a study with 1832 Chinese older adults observed a high agreement (*k* = 0.768) among these definitions [45]. Nevertheless, Chieng et al. [3] reported a fair agreement between these definitions in Malaysian T2DM patients (*k* = 0.229).

Similarly, a very good agreement (*k* = 0.859) was observed between NCEP-ATP III and IDF/Harmonized definitions. Both glucose and central obesity have different cut-off points between these definitions. Previous research in adults has shown high concordance between the NCEP-ATP III and IDF definitions in different countries like Mexico (*k* = 0.873) [46] or Turkey (*k* = 0.840) [47], while others found a moderate agreement in Indian (*k* = 0.440) [48] or Ethiopian (NCEP-ATP III vs. IDF: *k* = 0.540; NCEP-ATP III vs. Harmonized: *k* = 0450) [49] populations. Moreover, a very good agreement (*k* = 0.841) was found among NCEP-ATP III and IDF definitions in Chinese older adults [45].A fair agreement (*k* = 0.404) was obtained between the NCEP-ATP III and WHO definitions. This result is consistent since three of the five criteria have different cut-off points. Likewise, a moderate agreement (*k* = 0.511) was reported in Mexican adults [46], a fair agreement (*k* = 0.370) was observed in Turkish adults [47], and a weak agreement (*k* = 0.150) was obtained in Ethiopian adults [49], whereas Chieng et al. [3] reported a very good agreement (*k* = 0.875) in Malaysian T2DM patients.

The agreement and disparity in diagnosis of MetS between definitions is very important in order to be able to compare the obtained results between different studies. This agreement can vary according to specific age [45,46,48] or ethnicity [3,46,47,48,49] of the sample studied. Therefore, more studies are needed to assess how the degree of agreement between diagnoses changes depending on the specific population studied.

According to sensitivity results, the NCEP-ATP III, AHA/NHLBI, IDF, and Harmonized criteria seem more useful to diagnose MetS than the WHO criteria in older adults with decreased functional capacity. All definitions showed good specificity; therefore, these definitions appear to be useful to rule out MetS in older adults when they are truly negative.

So, it seems that all definitions can diagnose the same percentage of subjects with MetS, with the exclusion of WHO criteria. These results are especially essential for Spanish healthcare professionals, since when using any of the four diagnostic definitions, the proportion of patients diagnosed of MetS will be very similar. However, if they use the WHO definition, they will not diagnose at least half of the real cases. Similar results have been found in others studies [45,46,47], but no studies were found with a European or Spanish older population. Therefore, more studies are needed with this population to know which definitions can be used indistinctly in health centers.

### 4.4. Odds Ratios of Individual Components of Metabolic Syndrome

The ORs of individual components of MetS to develop MetS are related to the pathophysiology. The pathogenic mechanisms of MetS are complex. Currently, it is under debate whether MetS represents a cluster of different medical conditions or manifestations of a common pathogenic mechanism. Environmental, genetic, and lifestyle factors such as the consumption of excess calories and lack of PA have been considered as major contributors to the development MetS. Visceral adipose is a hormonally active tissue of body fat, which has specific biochemical characteristics that affect in different diseases, specifically, it has a main role in the implicated pathways in the development of MetS [50,51]. Moreover, insulin function is modified by a decreased expression of the glucose transporter 4 (GLUT4) gene [52]. This hormone is more affected by visceral adipose than subcutaneous fat, as visceral lipolysis leads to a rose supply of free fatty acids (FFAs) to the liver through splanchnic circulation. An increase in FFAs leads to increased TGs synthesis and the production of very low-density lipoproteins (VLDL) in the liver. This lipoprotein contains apolipoprotein-B, and its main function is transport TGs and cholesterol from the liver to peripheral tissues. Thus, an increase in low-density lipoprotein cholesterol and VLDL synthesis, and at the same time a reduction in HDL-C, are indirect effects of insulin resistance caused by altered lipid metabolism in the liver, increasing cardiovascular risk [51,53].

The present study has not focused on finding the etiology of MetS but shows results that help to understand this set of clinical signs. In this research it has been observed that participants with elevated TGs and reduced HDL-C have a high risk of developing MetS, which results logically from a pathophysiological point of view. In this way, it is known that participants with central obesity and visceral adiposity show increased TGs and glucose and reduced HDL-C [50]. However, this study has observed that central obesity [ORs = 18.0] is one of the main factors in the development of MetS, and that it is accompanied by elevated TGs (ORs = 37.5) and reduced HDL-C (ORs = 27.3), which are really the consequence of central obesity [50]. Therefore, the visceral adiposity and the alteration in lipid and glucose metabolism seem to explain the set of clinical signs of MetS. Finally, according to present results, hypertension does not seem to influence so much in the development of MetS (ORs = 4.8), but this result may be affected by tacking antihypertensive drugs. It seems that this criterion appears when lipid and glycolytic metabolic disorders are previously developed, since it could be secondary to the loss of the vasodilator effect of insulin and vasoconstriction caused by FFAs. Additional mechanisms include increased sympathetic activation and sodium reabsorption in kidneys [50].

### 4.5. Association between Frailty Status and Metabolic Syndrome

Contrary to expectations, this study did not find significant associations between frailty and MetS in older adults, since the ROB and FRA groups have a very similar prevalence of MetS, with the ROB group even being higher. The high prevalence of MetS in the ROB group could be explained by other modifiable factors such as diet, smoking, and alcohol intake, among others not considered for present study. The influence of age in each group was analysed, but no significant differences were found between groups. These ROB participants might have good functional capacity, but poor metabolic health that justifies these results. When the PREF and FRA groups were analysed, it seemed that MetS is more prevalent as frailty status increases. Similar results were obtained in a cross-sectional study involving older adults with MetS which analysed the prevalence of this syndrome according to frailty, and there were no significant differences in the prevalence of reduced HDL-C, central obesity, hypertension, and general prevalence of MetS between ROB, PREF, and FRA groups [2]. However, a cross-sectional study with older adults from Berlin observed that the ORs of being pre-frail or frail were significantly increased with decreased HDL-C (ORs: 1.5) and elevated WC (ORs: 1.65), whereas there were no associations with elevated TGs and glucose, and hypertension [54]. In the same way, obesity seems to be the main individual component of MetS and favors the decline of functional capacity [55]. Even though in obese people muscle mass may not be decreased, the function of muscle is damaged, specifically, in older adults. Therefore, the assessment of central obesity is crucial in older adults to identify subjects with higher risk of loss function [56].

On the other hand, the analysis of each criterion helps us to understand why the ROB group presents a prevalence of MetS similar to that of the FRA group, since they present the highest prevalence of elevated glucose and hypertension and the second of central obesity and reduced HDL-C. Therefore, subjects could maintain a good intrinsic capacity, but they could have a bad metabolic state. It is necessary to remember the great influence of individual genetics in the protective or facilitating factors of metabolic alterations [57,58]. In addition, in order to maintain a good intrinsic capacity, it is convenient to have a healthy diet, as the alteration at the metabolic level can be influenced to a large extent by eating habits.

### 4.6. Association between Physical Activity and Metabolic Syndrome

With respect to the last research question, an association between PA and MetS was found. Central obesity and hypertension prevalence were significantly lower in the active group than in the non-active group. However, there were no statistically significant differences in the prevalence of MetS between PA groups or in the HDL-C, glucose, and TGs levels. Moreover, it was observed that the whole active group and the active women group seemed to show that being active is associated with a lower risk of developing MetS, but not in men.

The role of PA in the development of MetS is not clear, since the results vary according to the studies [20]. There are variables such as age, sex, ethnicity, intensity of PA, or the method of measuring PA that can influence the results [10,11,17,20]. Previous studies reported similar results to the present research. In this way, a study showed that adults who maintain an active lifestyle have lower prevalence of MetS and its individual CVD risk factors [59]. Also, a study with older adults observed that the participant with high levels of PA had a lower risk of MetS (OR = 0.31, 95% CI = 0.13, 0.72) and more healthful levels of hypertension (OR = 0.39, 95% CI = 0.20, 0.77) and HDL-C (OR = 0.39, 95% CI = 0.18, 0.84) [60]. In the present study, it appears that the whole active group and the active women group have a lower risk of developing MetS. Moreover, a lower prevalence of central obesity and hypertension has been observed in the active group, whereas HDL-C and TGs levels did not differ from the non-active group. The results obtained in the active men group could be justified by a non-representative sample (*n* = 15). These different results could be influenced by the age of the sample and the intensity of PA performed. Another cross-sectional study of community-dwelling older men observed that higher levels of MVPA and lower levels of sedentary behaviour were associated with better metabolic health in terms of central obesity, insulin level, and MetS [61]. Finally, a systematic review measured the association between PA and MetS, and it concluded that PA had a significant association with MetS, varying in terms of strength and direction of association for the different parameters assessed [17]. It seems that central obesity is the only criterion of the MetS that can be significantly improved in active people. The associations between PA and HDL-C, TGs, hypertension, and glucose are not clear. Further studies are needed to know the independent contribution of PA to the overall risk of MetS.

Although the effects of PA on MetS are not clear, the role of exercise in MetS is shown in different studies. A meta-analysis observed that aerobic exercise improved WC −3.4 cm; glucose −0.15 mmol/L; HDL-C 0.05 mmol/L; TGs −0.29 mmol/L; DBP −1.6 mmHg; and cardiorespiratory fitness 4.2 mL/kg/min, among other outcomes [62]. Moreover, another meta-analysis compared the effects of drug and exercise interventions on health outcomes, and they reported that exercise and many drug interventions are often potentially similar in terms of their mortality benefits in the secondary prevention of coronary heart disease, rehabilitation after stroke, treatment of heart failure, and prevention of diabetes [63]. Therefore, exercise is an effective tool to decrease the prevalence of cardiometabolic risk factors of MetS in adults, and it could prevent the development of several health disorders such as CVD [8] or cancer [5].

### 4.7. Strengths and Limitations

To the best of our knowledge, this report is the first to investigate the prevalence of individual components, the concordance between the different definitions of MetS, and the associations between MetS, PA, and frailty in older adults with decreased functional capacity from a Spanish population. Secondly, a rigorous and systematic methodology was used to assess health outcomes through a structured questionnaire, physical examination, and biochemical analysis. Thirdly, it has been reported that the prevalence of MetS is modified according to the diagnostic criteria used, showing that IDF and Harmonized definitions can be used interchangeably in older adults. Fourthly, the importance of being physically active in older adults is highlighted.

Some potential limitations should be also mentioned. First, the research has not been able to analyse the association between MetS, CVD, T2DM, and cancer. These results would be interesting as a way to explain how MetS could increase the risk of these diseases. Second, even though our study has a considerable sample size to describe the prevalence of MetS in older people, especially those with symptoms of collapsing capacity, it is still reduced to accurately estimate the prevalence of MetS in Spanish older adults with decreased functional capacity. Thus, studies with a larger sample size should be developed to establish deeper conclusions. Third, there is a lack of adaptation of the diagnostic criteria for MetS to the Spanish and/or older adult population. It would be interesting to design adjusted criteria to this sample in order to know the real prevalence of MetS.

## 5. Conclusions

The prevalence of MetS changes according to the diagnostic criteria used, resulting with the lowest prevalence when utilizing the WHO definition and with the highest prevalence when utilizing the IDF and Harmonized definitions. No significant differences were observed in the prevalence of MetS between men and women. Central obesity is the most prevalent cardiometabolic risk factor of MetS in Spanish older adults with decreased functional capacity. The IDF and Harmonized definitions had the best agreement with the highest Kappa index. Individuals with elevated TGs, reduced HDL-C, and central obesity were at significantly elevated ORs to develop MetS in this sample. The prevalence of MetS according to frailty status did not show significant differences between groups. Finally, the present study shows that there is an association between PA and MetS, since central obesity and hypertension prevalence are significantly lower in the active group than in the non-active group. Furthermore, it seems that the whole active group and the active women group show a lower risk of developing MetS.

In future investigations, it would be interesting to analyse the effect of exercise interventions on MetS in Spanish older adults with decreased functional capacity. It seems that exercise could be an effective tool to reduce the incidence of MetS in the future. Moreover, it would be interesting to analyse a representative sample of older adults in Spain.

## Figures and Tables

**Table 1:** The WHO, NCEP ATP III, AHA/NHLBI, IDF, and Harmonized definitions of metabolic syndrome.

	WHO (1999)	NCEP-ATP III (2001)	AHA/NHLBI (2005)	IDF (2006)	Harmonized (2009)
Definitions	Insulin resistance plus 2 additional risk factors	≥3 of the following five risk factors:	≥3 of the following five risk factors:	Central obesity plus 2 other features	≥3 of the following five risk factors:
Elevated glucose	≥6.1 mmol/L (110 mg/dL) or diagnosed T2DM	≥6.1 mmol/L (110 mg/dL) or diagnosed T2DM	≥5.6 mmol/L (100 mg/dL) or diagnosed T2DM	≥5.6 mmol/L (100 mg/dL) or diagnosed T2DM	≥5.6 mmol/L (100 mg/dL) or diagnosed T2DM
Central obesity	Men: WHR > 0.9 and/or BMI >30 kg/m^2^	Men: WC > 102 cm	Men: WC > 102 cm	Men: WC > 94 cm or BMI > 30 kg/m^2^	Ethnic cut point for WC (Mediterranean, WC > 94 cm)
	Women: WHR > 0.85 and/or BMI >30 kg/m^2^	Women: WC> 88 cm	Women: WC > 88 cm	Women: WC > 80 cm or BMI > 30 kg/m^2^	Ethnic cut point for WC (Mediterranean, WC > 80 cm)
Hypertension	≥140/90 mmHg or treatment	≥130/85 mmHg or treatment	≥130/85 mmHg or treatment	≥130/85 mmHg or treatment	≥130/85 mmHg or treatment
Elevated TGs	≥1.7 mmol/L (150 mg/dL) or treatment	≥1.7 mmol/L (150 mg/dL) or treatment	≥1.7 mmol/L (150 mg/dL) or treatment	≥1.7 mmol/L (150 mg/dL) or treatment	≥1.7 mmol/L (150 mg/dL) or treatment
Reduced HDL-C	Men: <0.9 mmol/L (35 mg/dL) or treatment	Men: <1.03 mmol/L (40 mg/dL) or treatment	Men: <1.03 mmol/L (40 mg/dL) or treatment	Men: <1.03 mmol/L 40 mg/dL) or treatment	Men: <1.03 mmol/L 40 mg/dL) or treatment
	Women: <1.0 mmol/L (39 mg/dL) or treatment	Women: <1.29 mmol/L (50 mg/dL) or treatment	Women: <1.29 mmol/L (50 mg/dL) or treatment	Women: <1.29 mmol/L (50 mg/dL) or treatment	Women: <1.29 mmol/L (50 mg/dL) or treatment

AHA, American Heart Association; BMI, body mass index; HDL-C, high-density lipoprotein cholesterol; IDF, International Diabetes Federation; NCEP-ATP III, National Cholesterol Education Program–Third Adult Treatment Panel; MetS, metabolic syndrome; NHLBI, National Heart, Lung and Blood Institute; T2DM, type 2 diabetes mellitus; TGs, triglycerides; WC, waist circumference; WHR, waist-to-hip ratio; WHO, World Health Organization.

**Table 2:** Socio-demographic, frailty status, physical activity, anthropometrics and body composition, and blood serum biochemical and blood pressure characteristics of the subjects.

Characteristic	Men (*n* = 33) *n* (%) or Mean ± SD	Women (*n* = 77) *n* (%) or Mean ± SD	*p* Value
Socio-demographic			
Age (y.)	80.5 ± 6.2	80.7 ± 5.7	0.838
Fried- Frailty status			**0.015**
Robust	12 (36.4%)	11 (14.3%)	
Pre-frail	17 (51.5%)	49 (63.6%)	
Frail	4 (12.1%)	17 (22.1%)	
Physical activity *			0.830
Active	15 (57.7%)	30 (44.8%)	
Non-Active	11 (42.3%)	37 (55.2%)	
Anthropometrics and body composition			
Weight (kg)	**81.6 ± 15.0**	**70.2 ± 13.4**	**<0.001**
Height (cm)	**166.7 ± 6.7**	**153.8 ± 6.2**	**<0.001**
BMI (kg/m^2^)	29.2 ± 5.5	30.0 ± 5.7	0.581
Body fat (%)	**30.8 ± 6.1**	**39.5 ± 6.3**	**<0.001**
WC (cm)	**102.5 ± 12.7**	**92.7 ± 12.2**	**0.001**
Hip circumference (cm)	103.8 ± 9.4	105.1 ± 10.4	0.639
WHR	**0.96 ± 0.05**	**0.86 ± 0.07**	**<0.001**
Serum Biochemical			
Glucose (mg/dL)	103.6 ± 15.6	105.8 ± 29.6	0.696
HDL-C (mg/dL)	48.8 ± 17.2	55.1 ± 15.9	0.080
TGs (mg/dL)	124.0 ± 64.4	131.1 ± 53.6	0.567
Blood pressure			
SBP (mmHg)	136.0 ± 20.7	136.9 ± 15.6	0.803
DBP (mmHg)	73.9 ± 12.7	73.5 ± 9.4	0.858

BMI, Body mass index; DBP, diastolic blood pressure; HDL-C, high-density lipoprotein cholesterol; n, number of participants of the sample; SBP, systolic blood pressure; SD, standard deviation; TGs, triglycerides; WC, waist circumference; WHR, waist-to-hip ratio. Boldface indicates significant results. * The total sample to assess physical activity was 93 participants (Men = 26).

**Table 3:** Prevalence of metabolic syndrome and the percentage of participants (*n* = 110) with individual components of metabolic syndrome by the WHO, NCEP ATP III, AHA/NHLBI, IDF, and Harmonized criteria, respectively.

Components of MetS	WHO			NCEP-ATP III			AHA/NHLBI			IDF			Harmonized		
	Men	Women	*p* Value	Men	Women	*p* Value	Men	Women	*p* Value	Men	Women	*p* Value	Men	Women	*p* Value
Elevated glucose	10 (30.3%)	23 (29.9%)	0.929	10 (30.3%)	23 (29.9%)	0.929	16 (48.5%)	31 (40.3%)	0.275	16 (48.5%)	31 (40.3%)	0.275	16 (48.5%)	31 (40.3%)	0.275
Central obesity *	**16 (88.9%)**	**32 (57.1%)**	**<0.001**	16 (48.5%)	49 (63.6%)	0.115	16 (48.5%)	49 (63.6%)	0.115	**21 (63.6%)**	**66 (85.7%)**	**<0.001**	**21 (63.6%)**	**66 (85.7%)**	**<0.001**
Hypertension **	6 (18.2%)	9 (11.7%)	0.081	6 (18.2%)	9 (11.7%)	0.081	6 (18.2%)	9 (11.7%)	0.081	6 (18.2%)	9 (11.7%)	0.081	6 (18.2%)	9 (11.7%)	0.081
Elevated TGs	8 (24.2%)	25 (32.5%)	0.067	8 (24.2%)	25 (32.5%)	0.067	8 (24.2%)	25 (32.5%)	0.067	8 (24.2%)	25 (32.5%)	0.067	8 (24.2%)	25 (32.5%)	0.067
Reduced HDL-C	7 (21.2%)	11 (14.3%)	0.086	11 (33.3%)	29 (37.7%)	0.314	11 (33.3%)	29 (37.7%)	0.314	11 (33.3%)	29 (37.7%)	0.314	11 (33.3%)	29 (37.7%)	0.314
Total prevalence of MetS by sex	4 (22.2%)	10 (17.5%)	0.483	13 (39.4%)	25 (32.5%)	0.214	10 (30.3%)	28 (36.4%)	0.196	11 (33.3%)	30 (39%)	0.236	11 (33.3%)	30 (39%)	0.236
Total prevalence of MetS	14 (15.7%)			38 (34.5%)			38 (34.5%)			41 (37.3%)			41 (37.3%)		

AHA, American Heart Association; HDL-C, high-density lipoprotein cholesterol; IDF, International Diabetes Federation; NCEP-ATP III, National Cholesterol Education Program—Third Adult Treatment Panel; MetS, metabolic syndrome; NHLBI, National Heart, Lung, and Blood Institute; TGs, triglycerides; WHO, World Health Organization. Boldface indicates significant results. * The total sample for central obesity in WHO criteria are 89 participants (Men = 33). This is due to central obesity in WHO criteria is calculated with waist-to-hip ratio, and the hip circumference of some participants were not measured. However, the other definitions calculate central obesity only with waist circumference. ** A total of 66 (Women = 51) out of 110 participants are on treatment with an antihypertensive drug.

**Table 4:** Agreement and disparity in diagnosis of metabolic syndrome using the WHO, NCEP ATP III, AHA/NHLBI, IDF, and Harmonized criteria.

Criteria		NCEP-ATP III		Sensitivity (%)	Specificity (%)	Kappa Index	Agreement
		MetS	Non-MetS				
WHO (*n* = 89)	MetS	12	2	0.387	0.965	0.404	Fair
	Non-MetS	19	56				
AHA/NHLBI (*n* = 110)	MetS	34	4	1	0.947	0.918	Very good
	Non-MetS	0	72				
IDF * (*n* = 110)	MetS	34	7	1	0.908	0.859	Good
	Non-MetS	0	69				
Harmonized * (*n* = 110)	MetS	34	7	1	0.908	0.859	Good
	Non-MetS	0	69				

AHA, American Heart Association; IDF, International Diabetes Federation; MetS, metabolic syndrome; NCEP-ATP III, National Cholesterol Education Program—Third Adult Treatment Panel; NHLBI, National Heart, Lung, and Blood Institute; WHO, World Health Organization. * IDF and Harmonized definitions have a Kappa index of 1.000.

**Table 5:** Odds ratios of individual components of MetS to develop MetS by the NCEP-ATP III criteria.

Individual Components of MetS (Prevalence in the Whole Sample)	Yes/No	Prevalence of MetS within Each Group (%)	Odds Ratio (95% CI)	Kappa Index
Elevated glucose (30%)	Yes	75.8	15.4 (5.7–41.6)	0.564
	No	16.9		
Central obesity (65.5%)	Yes	50.0	18.0 (4.0–80.4)	0.369
	No	5.3		
Hypertension (13.6%)	Yes	66.7	4.8 (1.5–15.3)	0.226
	No	29.5		
Elevated TGs (33%)	Yes	84.8	37.5 (11.7–119.8)	0.689
	No	13.0		
Reduced HDL-C (42.7%)	Yes	86.8	27.3 (9.0–82.7)	0.638
	No	13.2		

CI, confidence intervals; HDL-C, high-density lipoprotein cholesterol; MetS, metabolic syndrome; NCEP-ATP III, National Cholesterol Education Program—Third Adult Treatment Panel; TGs, triglycerides.

## Data Availability

Not applicable.
